# Sat Down Upon

**Published:** 1892-05

**Authors:** 


### SAT DOWN UPON.

Our friend, Dr. Q. C. Smith, whom some call the watch dog of the Treasury, was in an unfortunate way at Tyler; all his resolutions were tabled; one of them, at least, we thought, unwisely. He called attention to the fact that railroads in Texas prohibit travel on all trains except regular passenger trains, and no matter how urgent a professional call to a distance may be, the physician has to wait for the regular train, greatly to the annoyance and inconvenience, no doubt, of many Texas doctors.

In light of this fact, he offered a resolution that the Texas State Medical Association memorialize and petition all the railroads to issue orders to permit doctors to ride on freight or any other train, at their own risk and by paying full fare.

What could have been more reasonable? And yet it was—tabled !

He offered another resolution, suggesting a change in the by-laws governing the election ot officers whereby the officers would be elected in open session—all hands voting; instead of by the nominating committee. Tabled.

The JOURNAL has repeatedly called attention to the fact that under our system the minority control the elections and run the

Association. A moment's reflection will show this: One representative from each county goes into the nominating committee, and has one vote. If he be from a county where there are only four or five or a dozen doctors, his vote represents that number of physicians, and weighs as much as that of the member from a county with hundreds of constituents, as Travis, for instance. Travis County Medical Association sends seven delegates, representing thirty or forty members. One only goes in the nominating committee, and hence the thirty or forty members of Travis County Medical Society have no more voice in elections than the most remote county on the frontier where there are almost no doctors. Is this right? or just?

We have pointed out at length how and why the doctor was sat down upon in the matter of his resolution looking to retrenchment. Dr. Smith will keep his seat in future, and let the Association run its own machine, he says.

And it is thus that members who really feel an interest in the Association, and would promote its best interests, are discouraged from any attempt to change the old order of things.

